# Influence of Sports Training in Foothills on the Professional Athlete’s Immunity

**DOI:** 10.3390/sports11020030

**Published:** 2023-01-30

**Authors:** Kristina A. Malsagova, Tatiana A. Astrelina, Evgenii I. Balakin, Irina V. Kobzeva, Elena Ya. Adoeva, Kseniya A. Yurku, Yuliya B. Suchkova, Alexander A. Stepanov, Alexander A. Izotov, Tatyana V. Butkova, Anna L. Kaysheva, Vasiliy I. Pustovoyt

**Affiliations:** 1 Biobanking Group, Branch of Institute of Biomedical Chemistry “Scientific and Education Center”, 109028 Moscow, Russia; 2 State Research Center—Burnasyan Federal Medical Biophysical Center of Federal Medical Biological Agency, 123098 Moscow, Russia; 3 S.M. Kirov Military Medical Academy, 194044 St. Petersburg, Russia

**Keywords:** physical activity, neuroplasticity, inflammation, immunodeficiency, athletes

## Abstract

Neuroplasticity and inflammation play important part in the body’s adaptive reactions in response to prolonged physical activity. These processes are associated with the cross-interaction of the nervous and immune systems, which is realized through the transmission of signals from neurotransmitters and cytokines. Using the methods of flow cytometry and advanced biochemical analysis of blood humoral parameters, we showed that intense and prolonged physical activity at the anaerobic threshold, without nutritional and metabolic support, contributes to the development of exercise-induced immunosuppression in sportsmen. These athletes illustrate the following signs of a decreased immune status: fewer absolute indicators of the content of leukocytes, lowered values in the immunoregulatory index (CD4^+^/CD8^+^), and diminished indicators of humoral immunity (immunoglobulins A, M, and G, and IFN-γ). These factors characterize the functional state of cellular and humoral immunity and their reduction affects the prenosological risk criteria, indicative of the athletes’ susceptibility to develop exercise-induced immunosuppression.

## 1. Introduction

Numerous neurobiological studies on the body’s adaptive reactions to stress have emphasized the significance of neuroplasticity and inflammation processes in the pathophysiology of subclinical changes. The adaptive stress response initially activates the sympathetic nervous system and hypothalamic-pituitary-adrenal (HPA) axis [1,2]. A myriad of concomitant homeostatic changes in the bodies of professional athletes contributes to the development of inflammatory processes caused by decreased absolute and relative counts of the B-cells and T-cells [3,4,5,6,7].

These processes are associated with the cross-interaction of the nervous and immune systems, which occurs via neurotransmitter signals, consequently impacting the adaptive immune responses. As a result of adaptive stress reactions in non-residential settings, the connection between nervous and immune systems changes, mainly in an immunosuppressive direction.

The hypothesis of our study is that the intense physical activity at the anaerobic threshold in hypoxic conditions without adjusted nutritional support and with incomplete recovery before the subsequent load contributes to the development of secondary sports immunodeficiency.

The aim is to study the details of the secondary immunodeficiency of foothill athletes depending on climatic conditions that contribute to the development of immunosuppression.

## 2. Materials and Methods

### 2.1. Demography

The voluntary participation of 49 male cyclic sports athletes was involved in this study (age: 25.7 ± 5.4 years, height: 182.9 ± 6.4 cm, body weight: 82.1 ± 9.9 kg). At the time of inclusion in the study, the athletes were considered healthy based on the results of a previously completed thorough medical examination, which included: instrumental examinations performed with fluorography, ultrasounds of the abdominal cavity and pelvic organs, echocardiography, electrocardiography, and an exercise stress test. Athletes did not perform high-intensity physical exercise 1 month prior to study enrollment.

### 2.2. Study Design

During the sports camp, 17 athletes who passed training camp in the Moscow region of Khimki, in the training center “Novogorsk”, were distributed into the first group (control); in the second group (experimental), 32 athletes training at the upper base, “Yug-Sport”, on the mountain Small Saddle, were included. During the study, the training center “Novogorsk” was characterized by a moderately continental climate: altitude 150 m above sea level, temperature—19 ± 3 °C, and relative humidity ranging from 29% to 94%. The upper base “Yug-Sport” mount Small Saddle is located at an altitude of 1242 m above sea level and is characterized by a moderately continental climate: the temperature—18 ± 2 °C, and relative humidity ranges from 47% to 92%. The main difference between the test groups was the altitude at which intensive training took place.

During the duration of the sports camps, the athletes did not take pharmacological preparations or biologically active additives that could affect the immune processes in their bodies.

**Figure 1:**
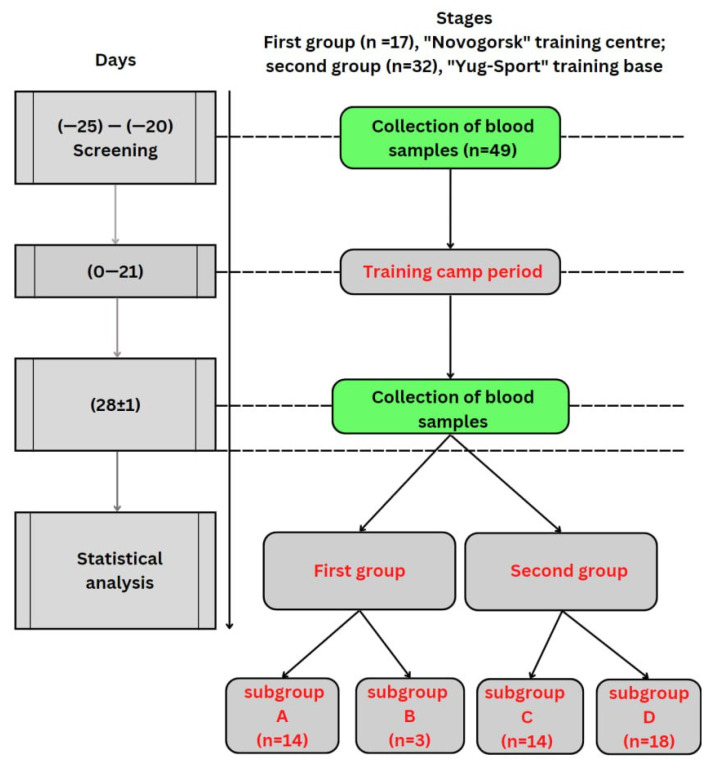
Scheme of the experimental design.

Physical activity was identical in both groups, twice a day for two hours, for 6 days a week for 21 days at the level of aerobic and anaerobic thresholds of 45 and 55%, respectively (Figure 1).

The presence of immunosuppression was recorded on the basis of the results of a decrease in the number of lymphocytes relative to their indicators of baseline examination for each athlete. At the end of the study, based on the criterion of immunosuppression, the differences in the frequency of occurrence of the symptom between the first and second groups were statistically assessed, which made it possible to compare the results of other indicators of the immune status of the examined athletes. After the primary analysis, four subgroups were identified in the first and second groups: the A and C had indicators equal to or higher than the background, and in the B and D subgroups the indicators were below the background (Tables 3–8, Section 3).

Blood samples were taken 20 days before the start of training camps and 7 days after they ended.

### 2.3. Laboratory

The indicators of cellular immunity and phagocytic activity were determined by flow cytometry using a BD FACSCanto II flow cytometer (Becton Dickinson, San Jose, CA, USA).The instrument was calibrated before measurements using BD™ Cytometer Setup and Tracking Beads Kit [8]. To determine the phagocytic activity of neutrophil granulocytes by measuring the respiratory (oxidative) burst after their stimulation with *E. coli* bacteria in human heparinized whole blood, a FagoFlowEx Kit (Exbio) was used according to the manufacturer’s instructions (https://www.exbio.cz/getattachment/7789d4a3-e051-4f4b-a12c-ef3398204df8/ED7042_IFU_v7_EN.pdf.aspx, accessed on 17 January 2023).

To determine the percentage of the main human lymphocyte subpopulations (Table 1), a BD Multitest™ IMK Kit (Becton Dickinson, San Jose, CA, USA) was used, according to the manufacturer’s instructions [9].

To study the phagocytic activity of granulocytes (Table 1), the PHAGOTEST reagent kit (Glycotope Biotechnology GmbH, Germany) was used according to the manufacturer’s instructions [10,11]. The results were analyzed with the BD FACSDiva™ software (version 6.0; Becton Dickinson, USA).

Humoral blood parameters (Table 2) were studied using the Cobas 6000 modular platform (Roche Diagnostics, Germany) [12].

After the completion of the training camp, athletes were divided into two groups based on their immune status indicators: the first group was characterized by the optimal variant of immune regulation, while the second group showed a decrease in immune parameters relative to the baseline examination.

### 2.4. Statistics

The obtained data were processed using the special application package STATISTICA v 13.1 (StatSoft, Inc., 2016, USA, Tulsa.), along with the Windows 2016 spreadsheet editor, Excel.

The data were described using the median and interquartile range values (the values of the first and third quartiles), as well as the mean and standard deviation. The groups were compared using a one-way analysis of variance (ANOVA). Additionally, a pairwise comparison was done, using a non-parametric Mann–Whitney U test. A chi-square test was used to compare the two groups. A correlation analysis was performed using Spearman’s rank correlation coefficient. During data processing, statistical significance was set at *p* < 0.05.

### 2.5. Ethics

Before the start of the experiment, the subjects were informed of the possible risks and inconveniences associated with all of the examination procedures, including blood sample collecting, echocardiography, and electrocardiography, after which each participant signed an informed consent form. The clinical and laboratory study was approved by the ethics committee of the Burnasyan Federal Medical Biophysical Center of the Federal Medical Biological Agency on 18 October 2018 (No. 10/2), in accordance with the Declaration of Helsinki.

## 3. Results

For decades, secondary immunosuppression has been considered a natural condition in athletes at the peak of their form [13,14,15]. New data may provide a different perspective on this process, as the functional state of cellular immunity is closely related to athletes’ environmental conditions and depends not only on the macrocycle period but also on the training camp location.

Among the first (training center “Novogorsk”) and the second (upper base “Yug-Sport”) groups of parallel training athletes, at the end of the study, 17.64% (3 athletes) and 56.25% (18 athletes), respectively, met the criterion for immunosuppression. Estimating frequencies using the chi-square test demonstrated a statistically significant (*p* < 0.05) difference between the first and second groups, which confirms the non-random nature of the distribution and the advisability of further comparison of the subgroups in each group.

Intense physical training under hypoxic conditions induced a decrease in peripheral blood absolute (Table 3 and Table 6) and relative (Table 4 and Table 7) levels of cellular and humoral (Table 5 and Table 8) immunity in the athletes. There was a significant decrease in immune functions in the repeated study, which is primarily related to the climatic features of the training process and, secondly, to the lack of adequate nutritional support in the groups.

The work determined the absolute and relative indicators of the cellular status of athletes in the control group (training center “Novogorsk”) (Table 3 and Table 4).

Additionally, for this group, indicators of humoral status were assessed (Table 5).

In the optimal functional state of the body, 20 days before the clinical and experimental study, the peripheral blood T-lymphocytes of the athletes were registered within the reference limits (Table 3, Table 4, Table 6, and Table 7). Since the examined individuals had a multidirectional immune response as a result of intense and prolonged physical activity under adverse environmental conditions, athletes were divided into four subgroups after completing the training camp, where A and C subgroups were characterized by optimal immune regulation, and B and D subgroups exhibited decreased immune indicators relative to the baseline examination. A decrease in the functional activity of cellular and humoral immunity was observed in B and D subgroups in comparison with A and C subgroups and background examination, where a decrease in the indices characterizing immune status and, consequently, decrease in functional status were registered in the athletes (Table 3, Table 4, Table 5, Table 6, Table 7 and Table 8).

According to Table 3, Table 4 and Table 5, it should be noticed that significant (*p* < 0.05) changes in the immune status in the examined individuals are recorded only in the absolute value of the number of lymphocytes in the blood stream, whereas in the sixth, seventh, and eighth tables, there are statistically significant changes in many indicators. These changes are associated with a disturbance of the adaptation mechanisms of athletes as a result of the impact of exogenous factors on the body during intensive physical training.

Indirect inhibition in CD3/4^+^ in the D subgroup was due to an increase in the proportion of anaerobic loads under hypoxic conditions. In this subgroup, there was also a decrease in CD3/8^+^, accompanied by a decrease in the cytolytic functions of the immune system (Table 6 and Table 7). Analysis of the absolute CD3/8^+^ values demonstrated a decrease in the total number of T-cells in the D subgroup compared to the C subgroup (Table 3). The obtained data confirm that the exacerbation of chronic diseases is caused by an increasing amount of physical exercise in conditions not typical for habitual residence. Reduced IFN-γ production in the D subgroup (Table 8) may also be associated with impaired immune homeostasis in the studied athletes.

## 4. Discussion

Intense physical training in adverse environmental conditions the in case of incomplete restoration of functional reserves results in the suppression of T-cells (CD3/4^+^, CD3/8^+^ and CD3/16/56^+^) (Table 6 and Table 7) in the D subgroup. Significant differences (*p* < 0.05) are recorded between the subgroups (Table 6 and Table 7) at re-examination as well as in the D subgroup compared to baseline. Significant (*p* > 0.05) shifts in CD3/4^+^ and CD3/8^+^ relative to immunosuppression in the C subgroup are observed in the D subgroup.

In the D subgroup, 5 of the 18 athletes showed signs of acute respiratory disease, which were apparently associated with uncompensated metabolic processes [16] that contribute to a decrease in the functional activity of the immune system and are characterized by nonspecific shifts in the cellular and humoral environment with weakened protective mechanisms in the body and the development of infectious disease [17].

The most important part in cellular metabolism is played by hypoxia-inducible factor 1-alpha (HIF-1α) [18], which has a leading function in the regulation of adaptive responses through the oxygen-sensitive pathway in CD3/8^+^ in response to high-intensity physical exercise in exogenous conditions [19,20,21,22]. However, this action in immune cells results in oxygen control in mRNA translation in the protein degradation pathway [23] and, consequently, to changes in T-cell phenotype [24]. In summary, gene expression when athletes are exposed to hypoxic hypoxia is associated with both stimulation and inhibition of the effector function of CD3/8^+^ T-cells. Consequently, the activation of hypoxia-induced signaling pathways in T-cells (CD3/8^+^) may depend on various exogenous factors that influence their immune activity.

In summary, it must be emphasized that transcriptional programs induced by HIF-1α play an important part in controlling gene expression [25] and functional variability of CD3/8^+^ in response to the development of hypoxic hypoxia in immune cells [24]. In addition, IFN-γ production is inhibited in T-cells (CD3/4^+^) as a result of reduced mitochondrial respiration during hypoxia (Table 8) [26].

To confirm the theory of HIF-1α-mediated regulation of adaptive reactions resulting in immunosuppression in athletes in response to intensive and prolonged training in foothill conditions, an additional study using metabolomic and proteomic complexes is required. A possible connection of stress-induced adaptive effects with the involvement of GVHD and the autonomic nervous system is not excluded, for which additional studies are also required for confirmation [9,27,28,29,30].

To confirm the reasons for the development of exercise-induced immunosuppression, a comprehensive study of the adaptive mechanisms of regulation in the body of athletes with an analysis of the metabolic activity of neurotransmitters, neuropeptides, and neurotrophic and immunological factors is necessary. Cellular metabolic pathways play a critical part in modulating immune functions [31]. The data obtained confirmed that in-tense physical activity is one of the most important factors linking metabolic and immune processes in the body.

Some authors have noted the possibility of cytotoxic effects resulting from intense physical activity at its peak without proper nutrient and metabolic correction, which are mainly mediated by different types of T-cells and is of fundamental importance in the immunological control of the development of immunosuppressive stress in the body of athletes [13,14,15,32,33].

In this context, T-lymphocytes are critical cells in the adaptive immune response, controlling the functional state of T-cells and fundamental to the strategy of maintaining the athlete at an optimal level of physical performance and functional state of the body.

T-cell-mediated immune responses are essential for the effective protection of athletes against infectious diseases [34]. This requires significant functional reserves, which contribute to the proliferation processes of T-cells (CD3/4^+^ and CD3/8^+^), NK cells, and B-cells.

Lack of immune activation of the transcriptional reprogramming of T-lymphocytes, necessary for the proliferation and differentiation of immune cells, results in the exacerbation of chronic and development of acute infectious diseases [17]. In this context, T-lymphocytes are critical cells in the adaptive immune response, which is fundamental to any strategy in immunotherapy or immunoprophylaxis.

The decreased expression of CD3/8^+^ in the D subgroup indicates impaired differentiation and functional activity of these cells, which is an important indicator of adaptive processes in the immune system [35,36,37,38]. In the literature, there are contradictory data on the expression of effector molecules by CD3/8^+^ cells under hypoxia conditions. For example, some authors state that there is an increase in IL-10 and IFN-γ production [19,39], while other researchers publish opposite results stating that hypoxia decreases IFN-γ production in T-cells [40].

It has recently been shown that chronic hypoxic exposure of the body contributes to T-cell depletion through mitochondrial dysfunction in immune cells [41,42,43]. An in-depth clinical and experimental study of the signaling pathways regulating the balance of immune cells sensitive to hypoxic hypoxia is required to confirm these findings.

Thus, when using nutrient–metabolic correction of highly qualified athletes during the training period under hypoxic conditions, it is necessary to consider the pathways of HIF-1α regulation and possible imbalance of neuroendocrine interaction, which contribute to the development of stress immunosuppressive action in the body of athletes.

In order to study the leading causes of the developing immunosuppressive effect of stress on the organism of the examined athletes of high-performance sports in detail, a comprehensive approach using genomic, proteomic, and metabolomic studies is necessary.

## 5. Conclusions

Observation of the second group demonstrated that intensive training under hypoxic conditions resulted in a significant (*p* < 0.05) decrease in cellular and humoral immunity parameters in 56.25% of the examined athletes, where nutritional and metabolic support for athletes from the beginning of an intensive training process under hypoxic conditions may help avoid future complications.

Signs of a decrease in the immune status are noted in the second group in 18 out of 32 examined athletes and are characterized by a significant (*p* < 0.05) decrease in absolute indicators of leukocytes—18.3%, lymphocytes—33.3%, T-lymphocytes—36, 4%, leukocytes/T-lymphocytes—23.5%, CD3/4^+^— 38.7%, CD3/8^+^— 37.2%, NK cells—33.3%, and CD19^+^— 22.5%, as well as the relative values of the immunoregulatory index CD4^+^/CD8^+^—by 6.5%, and indicators of humoral immunity (Ig A—14.3%, Ig M—12.1%, Ig G—8. 1%, and IFN-γ—29.3%).

The most significant (*p* < 0.05) prenosological risk criteria for the development of sports immunosuppression (decreases in absolute indicators: lymphocytes, T-lymphocytes, CD3/4^+^, CD3/8^+^, and NK cells), characterizing the functional state of cellular and humoral immunity, are distinguished among the listed indices.

In the study of sports immunosuppression, to clearly understand the specific nature of metabolic dysregulation in immune cells during exacerbation of chronic diseases in athletes, studies in the field of immunometabolism can be considered the most promising. New data will provide an opportunity to develop additional pharmacological tools for both individual and combined use with standard approaches in nutritional–metabolic support.

## Figures and Tables

**Table 1:** Reference values characterizing the immune status in terms of cellular parameters.

Cell Type	Norm (%)	Norm (10^9^/L)
Leukocytes	-	4.0–9.0
Lymphocytes	19.0–37.0	1.2–3.0
Stab neutrophils	1.0–6.0	-
Segmented neutrophils	47.0–72.0	-
Eosinophils	0.5–5.0	0.1–0.3
Basophils	0–1.0	0–0.07
Monocytes	3.0–11.0	0.1–0.9
T-lymphocytes CD45^+^ CD3^+^	55.0–80.0	0.95–1.8
T-helpers CD45^+^ CD3^+^ CD4^+^	31.0–51.0	0.57–1.1
T-killers (CTL) CD45^+^ CD3^+^ CD8^+^	19.0–35.0	0.45–0.85
NK cells CD45^+^ CD3^−^ CD(16 + 56)^+^	7.0–20.0	0.18–0.42
B- lymphocytes CD45^+^ CD3^−^ CD19^+^	6.0–19.0	0.15–0.4
Index CD4^+^/CD8^+^	1.5–2.0	-
Phagocytic activity of blood granulocytes	55.0–95.0	-

**Table 2:** Reference values characterizing the immune status by humoral parameters.

Parameter	Norm	Measure
Immunoglobulin class G (IgG)	7.0–16.0	g/L
Immunoglobulin class A (IgA)	0.7–4.0	g/L
Immunoglobulin class M (IgM)	0.4–2.3	g/L
Immunoglobulin class E (IgE)	1.31–165.3	IU/mL
Interferon alpha (IFN-α)	640.0–1280.0	IU/mL
Interferon gamma (IFN-γ)	128.0–256.0	IU/mL

**Table 3:** Absolute parameters of the cellular status of athletes in the first (control) group (training center “Novogorsk”) throughout the study.

Parameters	Baseline Examination, (n = 17)	Day 7, Subgroup A (n = 14)	Day 7 Subgroup B (n = 3)
Leukocytes (10^9^/L)	6.9 [6.3–7.6]	7.3 [6.2–8.4]	6.2 [3.9–8.5]
Lymphocytes (10^9^/L)	2.2 [2.1–2.4]	2.4 [2.1–2.6] *^,^**	1.8 [1.7–1.9] *^,^**
T-lymphocytes (10^9^/L)	1.6 [1.4–1.7]	1.7 [1.5–1.9]	1.2 [0.7–1.7]
Leukocytes/T-lymphocytes (10^9^/L)	4.5 [4.0–5.0]	4.3 [3.6–5.0]	5.1 [4.2–5.6]
CD3/4^+^, (10^9^/L)	0.9 [0.9–1.0]	1 [0.9–1.1]	0.7 [0.4–1.1]
CD3/8^+^, (10^9^/L)	0.6 [0.5–0.7]	0.6 [0.5–0.8]	0.4 [0.2–0.6]
CD19^+^, (10^9^/L)	0.3 [0.2–0.3]	0.3 [0.2–0.4]	0.2 [0–0.4]
CD16/56^+^, (10^9^/L)	0.4 [0.3–0.4]	0.4 [0.3–0.4]	0.3 [0.2–0.6]
CD3/16/56^+^, (10^9^/L)	6.9 [6.3–7.6]	7.3 [6.2–8.4]	6.2 [3.9–8.5]

*–*p* < 0.05 changes are significant when comparing A and B subgroups; **–*p* < 0.05 changes are significant relative to the baseline examination; [Q1–Q3]–non-parametric descriptive statistics (non-normal distribution), M–median, Q1–lower quartile (25%), Q3–upper quartile (75%).

**Table 4:** Relative indicators of the cellular status of athletes in the first (control) group before and after sports camp (training center “Novogorsk”).

Parameter	Baseline Examination (n = 17)	Day 7, Subgroup A (n = 14)	Day 7 Second Subgroup B (n = 3)
Stab neutrophils (%)	53.8 [50.5–57.2]	53.4 [47.4–59.3]	56.3 [46.3–66.2]
Segmented neutrophils (%)	2.4 [1.8–3.1]	2.1 [1.2–2.9]	4.0 [1.6–5.6]
Eosinophils (%)	0.7 [0.6–0.9]	0.7 [0.5–0.9]	0.6 [0.2–1.1]
Basophils (%)	9.7 [8.8–10.6]	9.7 [8.2–11.1]	9.7 [1.6–17.8]
Monocytes (%)	32.8 [30.1–35.4]	33 [28.7–37.3]	32.1 [23.1–41.0]
Lymphocytes (%)	71.2 [68.8–73.6]	71.6 [68.5–74.7]	68.1 [41.9–94.3]
CD3^+^, (%)	42.1 [39.6–44.7]	42.0 [38.4–45.5]	40.8 [19.6–61.8]
CD3/4^+^, (%)	26.2 [24–28.5]	26.8 [23.1–30.4]	23.4 [13.8–33.1]
CD3/8^+^, (%)	1.7 [1.52–1.9]	1.7 [1.3–2.0]	1.7 [0.96–2.53]
Index CD4/CD8^+^ (%)	12.0 [10.6–13.4]	13.2 [10.6–15.8]	11.3 [3.7–18.9]
CD19^+^ (%)	16.1 [13.4–18.7]	14.5 [11.0–18.0]	19.0 [10.0–33.8]
CD16/56^+^ (%)	52.0 [43.6–60.5]	50.5 [41.9–59.1]	58.8 [37.9–85.3]
CD3/16/56^+^ (%)	53.8 [50.5–57.2]	53.4 [47.4–59.3]	56.3 [46.3–66.2]
Phagocytic activity of blood granulocytes (%)	2.4 [1.8–3.1]	2.1 [1.2–2.9]	4.0 [1.6–5.6]

[Q1–Q3]—non-parametric descriptive statistics (non-normal distribution), M—median, Q1—lower quartile (25%), Q3—upper quartile (75%).

**Table 5:** Parameters of the humoral status of athletes in the first (control) group before and after sports camp (training center “Novogorsk”).

Parameter	Baseline Examination (n = 17)	Day 7, Subgroup A (n = 14)	Day 7 Subgroup B (n = 3)
IgG (g/L)	11.8 [11.0–12.6]	11.4 [10.2–12.6]	15.2 [9.9–17.5]
IgA (g/L)	2.2 [2.0–2.4]	2.3 [1.9–2.6]	2.1 [1.5–2.4]
IgM (g/L)	1.0 [0.92–1.2]	1.0 [0.87–1.15]	1.0 [0.6–1.5]
IgE (IU/mL)	69.3 [44.0–91.0]	35.2 [17.5–52.8]	36.2 [7.9–50.6]

[Q1–Q3]—non-parametric descriptive statistics (non-normal distribution), M—median, Q1—lower quartile (25%), Q3—upper quartile (75%).

**Table 6:** Absolute parameters of the cellular status of athletes in the second (experimental) group (upper base “Yug-Sport”) before and after sports camp.

Parameters	Baseline Examination (n = 32)	Day 7, Subgroup C (n = 14)	Day 7 Subgroup D (n = 18)
Leukocytes (10^9^/L)	7.0 [6.4–7.7]	8.2 [7.6–8.9] *^,^**	6.7 [6.1–7.5]*
Lymphocytes (10^9^/L)	2.3 [2.1–2.6]	3.0 [2.8–3.3] *^,^**	2 [1.7–2.2] *^,^**
T-lymphocytes (10^9^/L)	1.7 [1.6–1.9]	2.2 [2–2.4] *	1.4 [1.2–1.5] *^,^**
Leukocytes/T-lymphocytes (10^9^/L)	4.3 [3.8–4.6]	3.9 [3.5–4.3] *	5.1 [4.6–5.5] *^,^**
CD3/4^+^, (10^9^/L)	1.0 [0.9–1.1]	1.2 [1.1–1.3] *^,^**	0.8 [0.7–0.8] *^,^**
CD3/8^+^, (10^9^/L)	0.6 [0.5–0.7]	0.9 [0.8–0.9] *^,^**	0.5 [0.5–0.6] *
CD19^+^, (10^9^/L)	0.3 [0.3–0.4]	0.4 [0.3–0.5] *	0.3 [0.29–0.34] *
CD3/16/56^+^, (10^9^/L)	0.3 [0.2–0.3]	0.3 [0.3–0.4] *	0.2 [0.2–0.3] *

*—*p* < 0.05 changes are significant when comparing the C and D subgroups; **—*p* < 0.05 changes are significant relative to the baseline examination; [Q1–Q3]—non-parametric descriptive statistics (non-normal distribution), M—median, Q1—lower quartile (25%), Q3—upper quartile (75%).

**Table 7:** Relative indicators of the cellular status of athletes in the second (experimental) group before and after sports camp (upper base “Yug-Sport”).

Parameter	Baseline Examination (n = 32)	Day 7, Subgroup C (n = 14)	Day 7 Subgroup D (n = 18)
Stab neutrophils (%)	53.5 [50.4–56.6]	51.4 [47.4–55.3] *	57.4 [55.2–61.6] *
Segmented neutrophils (%)	2.4 [1.7–3.2]	3.0 [2.1–3.9]	2.8 [1.8–3.6]
Eosinophils (%)	0.2 [0–0.3]	0.3 [0.2–0.5]	0.2 [0–0.3]
Basophils (%)	8.8 [8.2–9.5]	8.5 [7.9–9.1]	8.4 [7.7–9.1]
Monocytes (%)	34.4 [31.1–37.6]	35.7 [33.3–42.3] *	28.9 [26.4–31] *^,^**
Lymphocytes (%)	73.3 [70.5–76.1]	73.6 [71.8–75.4]	71.0 [69–72.9]
CD3^+^, (%)	42.7 [40.7–44.6]	41.5 [39.5–43.5] *	38.9 [37–40.7] *^,^**
CD3/4^+^, (%)	26.4 [25.0–27.8]	27.9 [26.3–29.5]	27.2 [25.4–28.9]
CD3/8^+^, (%)	1.7 [1.5–1.8]	1.6 [1.4–1.8]	1.5 [1.4–1.7]
Index CD4/CD8^+^ (%)	14.0 [12.6–15.4]	14.2 [12.8–15.5] *	17.0 [15.6–18.5] *^,^**
CD19^+^ (%)	10.3 [8.5–12.1]	10.4 [8.7–12.1]	10.8 [9.4–12.2]
CD16/56^+^ (%)	66.3 [62.0–70.4]	63.7 [59.8–67.7]	64.4 [59.9–68.8]
CD3/16/56^+^ (%)	53.5 [50.4–56.6]	51.4 [47.4–55.3] *	57.4 [55.2–61.6] *
Phagocytic activity of blood granulocytes (%)	2.4 [1.7–3.2]	3.0 [2.1–3.9]	2.8 [1.8–3.6]

*—*p* < 0.05 changes are significant when comparing the C and D groups; **—*p* < 0.05 changes are significant relative to the baseline examination; [Q1–Q3]—non-parametric descriptive statistics (non-normal distribution), M—median, Q1—lower quartile (25%), Q3—upper quartile (75%).

**Table 8:** Parameters of the humoral status of athletes in the second (experimental) group before and after sports camp (upper base “Yug-Sport”).

Parameter	Baseline Examination (n = 17)	Day 7, Group C (n = 14)	Day 7 Group D (n = 3)
IgG (g/L)	11.0 [10.3–11.7]	11.1 [10.4–11.7]	10.2 [9.5–10.8]
IgA (g/L)	1.5 [1.2–1.7]	1.4 [1.2–1.6]	1.2 [0.9–1.5]
IgM (g/L)	1.0 [0.9–1.12]	0.91 [0.79–1]	0.8 [0.7–1]
IgE (IU/mL)	69.3 [44.0–91.0]	77.0 [47.7–96.4] *	36.1 [24.8–47.3] *
IFN-α (IU/mL)	753.0 [585.0–921.0]	582.0 [471.0–693.0]	567.0 [447.0–714.0]
IFN-γ (IU/mL)	111.3 [95.9–128.1]	122.0 [103.6–139.3] *	86.1 [76.3–95.2] *^,^**

*—*p* < 0.05 changes are significant when comparing the C and D subgroups; **—*p* < 0.05 changes are significant relative to the baseline examination; [Q1–Q3]—non-parametric descriptive statistics (non-normal distribution), M—median, Q1—lower quartile (25%), Q3—upper quartile (75%).

## Data Availability

Not applicable.
